# The effectiveness and safety of conservative interventions for positional plagiocephaly and congenital muscular torticollis: a synthesis of systematic reviews and guidance

**DOI:** 10.1186/s12998-020-00321-w

**Published:** 2020-06-11

**Authors:** Julie Ellwood, Jerry Draper-Rodi, Dawn Carnes

**Affiliations:** 1 University College Osteopathy, 275 Borough High St, London, SE1 1JE England; 2 Faculty of Health, University Applied Sciences and the Arts Western Switzerland, Fribourg, Switzerland

**Keywords:** Paediatric, Positional plagiocephaly, Congenital muscular torticollis, Systematic review

## Abstract

**Aim:**

To investigate for congenital muscular torticollis (CMT) and positional plagiocephaly (PP) the effectiveness and safety of manual therapy, repositioning and helmet therapy (PP only) using a systematic review of systematic reviews and national guidelines.

**Methods:**

We searched four major relevant databases: PubMed, Embase, Cochrane and MANTIS for research studies published between the period 1999–2019. Inclusion criteria were systematic reviews that analysed results from multiple studies and guidelines that used evidence and expert opinion to recommend treatment and care approaches. Three reviewers independently selected articles by title, abstract and full paper review, and extracted data. Selected studies were described by two authors and assessed for quality. Where possible meta-analysed data for change in outcomes (range of movement and head shape) were extracted and qualitative conclusions were assessed.

**Results:**

We found 10 systematic reviews for PP and 4 for CMT. One national guideline was found for each PP and CMT. For PP, manual therapy was found to be more effective than repositioning including tummy time (moderate to high evidence) but not better than helmet therapy (low evidence). Helmet therapy was better than usual care or repositioning (low evidence); and repositioning better than usual care (moderate to high evidence).

The results for CMT showed that manual therapy in the form of practitioner-led stretching had moderate favourable evidence for increased range of movement. Advice, guidance and parental support was recommended in all the guidance to reassure parents of the favourable trajectory and nature of these conditions over time.

**Conclusions:**

Distinguishing between superiority of treatments was difficult due to the lack of standardised measurement systems, the variety of outcomes and limited high quality studies. More well powered effectiveness and efficacy studies are needed. However overall, advice and guidance on repositioning (including tummy-time) and practitioner-led stretching were low risk, potentially helpful and inexpensive interventions for parents to consider.

**Systematic review registration number:**

PROSPERO 2019 CRD42019139074.

## Introduction

Congenital muscular torticollis (CMT) is a postural, musculoskeletal deformity evident at, or shortly after, birth. It results from unilateral shortening and increased tone of the sternocleidomastoid (SCM) muscle and presents as lateral flexion of the head to the ipsilateral side with rotation to the contralateral side [1]. It is potentially a painful condition for infants and can present with a pseudotumor in the SCM muscle [2]. It is the third most common congenital musculoskeletal condition in newborns with an incidence ranging from 0.3 to 16% [3]. CMT has been associated with dysfunction in the upper cervical spine and is sometimes referred to as kinetic imbalance due to subocciptal strain (KISS) [2]. Treatment approaches for CMT include manual therapy (including practitioner-led stretching exercises) [4], repositioning therapy (including tummy time) [1] and, in severe non-resolving cases, botulinum and surgery [5]. CMT can lead to secondary changes such as cranial asymmetry, and also to functional problems, including breastfeeding problems [2].

Cranial asymmetry, also known as plagiocephaly, is the most common form of ‘flat head syndrome’ and presents itself as an asymmetrical head shape. Positional plagiocephaly (PP) (sometimes referred to as deformational plagiocephaly or non-synostotic plagiocephaly) typically occurs in infants and results from mechanical factors which, when applied over a period of time in utero, at birth, or postnatally, alter the shape of the skull [6]. In this condition there is flattening of one side of the occiput, with anterior displacement of the ipsilateral ear. The region of occipital flattening relates to the side that the head is toward when in the supine sleeping position [7].

A rise in the prevalence of PP occurred after widespread implementation in western countries of the ‘Back to Sleep’ campaigns which recommended that healthy term infants be positioned on their backs during sleep [8] to prevent Sudden Infant Death Syndrome (SIDS). Prior to 1992, the incidence of the deformity was estimated at 1 in 300 infants [9]. Estimates for PP prevalence now range from 16 to 48% of typical healthy infants younger than 1 year depending on the diagnostic criteria used and 37.8% for infants aged between 8 and 12 weeks old [10]. Considering this large increase in the incidence of PP, there has been much interest in investigating, managing, and preventing this condition [7]. Although the optimal timing and modality of interventions have yet to be clearly established, primary treatments for plagiocephaly are nonsurgical and include observation, head repositioning, manual therapy (including practitioner-led stretching exercises), and helmet therapy/orthotic devices [8].

Head repositioning therapy is usually performed by the parents with the purpose of positioning the infant’s head on the non-flattened side and includes advice on ‘tummy time’ recommendations. Helmet therapy, sometimes referred to as orthotics or moulding therapy, generally uses a plastic helmet with the aim of reshaping the deformed skull to a normal shape without restricting the cranial growth [11, 12]. Manual therapies such as that given by chiropractors, osteopaths, physiotherapists and physical therapists centre on techniques administered passively to the infant either through cervical articulation and soft tissue muscle stretching to restore neck function and/or soft tissue tension release/reduction [13]. Manual therapies also include practitioner-led stretching exercises, whether administered directly by a practitioner or by a practitioner guided parent.

The relationship between CMT and PP is slightly unusual as causality can go in either direction and infants can suffer from both conditions at the same time. There are similarities between the management strategies for both PP and CMT but limited research exists on their effectiveness. In addition, clear treatment protocols which take severity of the condition and age of the infant into consideration are lacking. There is concern about both CMT and PP due to their association with developmental dysplasia of the hip, brachial plexus injury, foot or lower limb anomalies and cognitive and motor development [1, 14].

## Aim

To investigate for congenital muscular torticollis (CMT) and positional plagiocephaly (PP) the effectiveness and safety of manual therapy, repositioning and helmet therapy (PP only) using a systematic review of systematic reviews and national guidelines.

## Methods

We conducted a systematic review of systematic reviews and clinical guidelines for conventional treatments of both PP and CMT. We reviewed existing analyses of information whether it was based on compiled qualitative or narrative analyses, meta-analysis or guideline consensus review (PROSPERO registered number: CRD42019139074). We used the PRISMA statement to guide the structure of this review [15].

### Definitions and outcomes of interest

#### Eligibility criteria

We included reviews that reported a systematic review methodology with more than one reviewer indicated in the review process. Literature reviews and editorials were not included.

We included guidelines where clear methodological procedures for development were reported. The guidance needed to be developed from systematically designed evidence reviews and expert panel consensus. The guidance must be intended for broad use at a national level rather than intended as guidance for a single clinic, hospital or a specific setting. We excluded guidance targeted at parents.

Only systematic reviews and national guidelines published in English within the last 20 years (1999–2019) were reviewed. Details on the eligibility criteria in Table 1.

**Table 1:** Eligibility criteria

• Population: children up to 12 months with PP and/or CMT • Interventions: Manual Therapy (including practitioner-led stretching exercises); Repositioning therapy (including tummy time); and Helmet therapy • Comparators: no limits • Outcomes: o reported outcome measures changes (for PP and CMT) o change in head shape irrespective of outcome measures used (for PP) o range of motion irrespective of outcome measures used (for CMT) • Study design: Systematic reviews and clinical guidelines	

##### Positional plagiocephaly

PP was defined as cranial asymmetry due to moulding and not to any other pathophysiological condition in infants who are otherwise healthy and thriving [3].

The outcomes of interest for PP were those reported in the included systematic reviews, including adverse events, for manual therapy (including practitioner-led stretching exercises), helmet therapy and repositioning therapy.

##### Congenital muscular torticollis

CMT was defined as asymmetrical muscular tension in the neck causing directional head preference from birth but present in infants up to 1 year old who were otherwise healthy and thriving [11]. The outcomes of interest for CMT were those reported in the included systematic reviews for symmetry/range of movement and adverse events. Interventions of interest were manual therapy (including practitioner-led stretching exercises) and repositioning therapy (including ‘tummy time’).

We defined manual therapy as any predominantly touch-based therapy administered by a trained and registered manual therapist, such as a chiropractor, osteopath, osteopathic physician, physical therapist or physiotherapist. We also included practitioner-led stretching exercises, whether administered directly by the practitioner or by a practitioner guided parent in our definition.

### Information sources

For systematic reviews we searched PubMed, MANTIS, Embase, and Cochrane databases. We searched the central clearing guideline database and known national health service centres for national guidelines developed for the treatment management and care of infants with PP and or CMT in English speaking countries (UK, Ireland, USA, Canada, Australia, New Zealand). The searches were conducted in June 2019 and we included all papers found from citation tracking up to this date.

### Search

Key search terms were: infant*, paediatric, pediatric and ‘nonsynostotic cranial deformity’, ‘nonsynostotic posterior plagiocephaly’, ‘positional plagiocephaly’, ‘congenital muscular torticollis’, ‘congenital torticollis’ and Treatment, ‘Manual therapy’, osteopath*, chiropract*, physiotherap* and derivatives automated by the search engines. Search strings are shown in the Additional file 1.

### Study selection

Results from searches on each database were downloaded into a reference management software: Endnote (version X4.0.2), and duplicates were removed. Titles and abstracts were screened by two independent researchers. Decisions of inclusion/exclusion of the articles were made in a meeting with the three authors to increase the consistency of the application of the inclusion and exclusion criteria. Citation tracking was used to triangulate our searches and check for missing reviews and guidance, as well as to identify other articles that may have not been indexed on PubMed. Full text papers were obtained for those that met the inclusion criteria and for those where it was unclear whether or not the abstract and title met the inclusion criteria. Inclusion and exclusion criteria were applied to all titles, abstracts and full papers. If more recent or updated version of guidelines than those gathered from the search were available, the more recent ones replaced those initially found.

Only guidance developed using clear methodological protocols for use at a national or international level were considered.

### Quality appraisal

We appraised the quality of the systematic reviews using a modified version of the AMSTAR 2 critical appraisal tool [16]. We used AMSTAR guidelines and agreed on our modified tool in advance of quality appraisal. Of the 15 quality categories assessed, 8 were selected by the authors for the final calculation quality, we allocated a score of 1 for Yes, 0 for No and 0.5 for Partial Yes. Final scores for the quality assessment had a range from 0 to 8 (8 being highest quality). Each study was appraised by 2 independent reviewers and a third reviewer was used if mitigation was required (please see Additional file 2).

We reported on the quality of the guidelines reviewed by using a modified version of the AGREE II framework [17]. We used AGREE II guidelines and agreed on our modified tool in advance of quality appraisal. We reflected on the procedures for development of the guidelines and specifically whether they were consensus driven and/or evidence review driven. We adapted the AGREE II scoring system as some guideline groups have supporting methodological manuals as opposed to a methods section in their publications or included online as part of the clinical guidance. The AGREE II checklist has 23 items evaluating 6 domains. Where more than 75% of the 23 items (> 17/23) were evaluated, considered and reported in the development of the guideline, we rated these as high-quality guidance. In guidance, where there was insufficient information to record a verdict, we left these domains blank. Guidelines of 16 or less domains did not receive an evaluation of high quality. Each guideline was appraised by one reviewer (please see Additional file 3 and 4).

### Data extraction and items

We designed a form to extract systematic reviews’ characteristics and data (see Tables 2 and 3). We summarised studies by type of intervention, number of studies included, number of participants, outcomes of interest and measure used. Where possible we extracted data on cranial asymmetry/head shape for PP and on passive cervical spine range of movement for CMT. We conducted a narrative synthesis review of outcomes where necessary and in the absence of synthesised data. We also extracted adverse event incident data and compared risk ratio between treatments where possible (see Tables 4 and 5).

**Table 2:** Characteristics of systematic reviews on Plagiocephaly

Authors	Participants/n/age/gender/condition (where reported)	Intervention (Conservative-repositioning, helmet, MT)	Timing of intervention	Number and type of studies included in review	Method of data synthesis (qualitative/meta-analysis)
Baird et al. 2016 [8]	Paediatric (< 18 years of age) patients with non-synostotic plagiocephaly or brachycephaly.	Manual Therapy	NR	3/ 2 x RCT, 1x prospective	Narrative
Klimo et al. 2016 [18]		Repositioning	NR	3 RCT’s (Class I), 1 prospective cohort study (Class II), and 6 retrospective cohort studies (Class III)	Narrative
Tamber et al. 2016 [19]		Helmet therapy	NR	1 prospective randomized controlled trial (Class II), 5 prospective comparative studies, (Class II), and 9 retrospective comparative studies (Class II).	Narrative
Bialocerkowski et al. 2005 [20]	Children < 12 months/PP	Positioning Vs Helmet + Manual therapy	NR	16/ 12 case series, 4 comparative studies	Narrative
Goh et al. 2013 [21]	Children/PP	Helmet therapy	NR	36/ 21 were primary research literature articles, 12 reviews, 2 letters, 1 methodology descriptor	Narrative
McGarry et al. 2008 [22]	Infants/PP	Helmet therapy	NR	20/ 3 reviews, 8 measurement, 9 mixed research method	Narrative
Paquereau, J. 2013 [23]	Children < 18 months/*n* = 1724 in original articles/PP	Orthotics Vs Repositioning	1-15 months	18/ 6 literature reviews, 12 original articles	Narrative
Parnell Prevost et al. 2019 [13]	Infants/0–12 weeks/paediatric conditions including cranial asymmetry	Manual Therapy	NR	50/ 32 RCT’s and 18 Observational studies	Narrative
Shweikeh et al. 2013 [7]	Children 3-18mths/PP	Repositioning Vs Helmet Vs Manual therapy	Long term	15/ 2 RCT’s, 4 case controls, 4 retrospective studies, 2 prospective studies, 2 longitudinal, 1 cross-sectional	Narrative
Xia et al. 2008 [11]	Healthy infants < 12 months with PP	Moulding helmet therapy vs head repositioning therapy	6 months	7 cohort studies	Narrative

**Table 3:** Characteristics of studies for Manual Therapy intervention for Congenital Muscular Torticollis

Authors	Participants/n/age/gender/condition	Timing of intervention	Number and type of studies included in review	Method of data synthesis (narrative/meta-analysis)
Brand et al., 2005 [24]	Children 0–23 months/KISS with PP, positional preference and infantile colic	NR	2 RCT’s	Meta-analysis
Driehuis et al., 2019 [4]	Infants and children/ paediatric conditions including CMT	8 weeks for CMT	26/12 RCT’s, 9 observational and 5 case reports 1 RCT related to CMT (Haugen et al., 2011)	Meta-analysis
Heidenreich et al., 2018 [25]	Infants and children/ CMT/ < 12 months	3 weeks with < 6-month FU where reported	20/ 10 described/ 4 retrospective cohorts, 2 Cohorts, 4 RCT’s	Narrative
Parnell Prevost et al., 2019 [13]	Infants/0–12 weeks/paediatric conditions including CMT	NR	50/ 32 RCT’s and 18 Observational 1 RCT related to CMT (Haugen et al., 2011)	Narrative

*FU* follow-up; *MT* manual therapy; *KISS* kinetic imbalance suboccipital strain syndrome *NR* not reported; *RCT* randomised controlled trial

**Table 4:** Summary of narrative analyses of treatments for Plagiocephaly

Author	Vs Control	Follow-up time-point	Cranial asymmetry – measurement method	Effect	Level of evidence	Amstar
**Helmet therapy**						
Goh et al. 2013 [21]	NR	NR	Anthropometric assessment	Inconclusive	Low	2
McGarry et al. 2008 [22]	NR	Up to 13 months	Bands, anthropometric callipers, moulding ring, observation, photography	Favourable	Low	4.5
Paquereau et al. 2013 [23]	Repositioning		Cranial index, CVAI, 3D, visual	Favourable	Low	3.5
Shweikeh et al. 2013 [7]	Repositioning and usual care	> 12 months	NR	Favourable in older children	NR	2
Xia et al. 2008 [11]	Repositioning only	6 months	NR (one study Helmet ×1.3 greater than repositioning)	Favourable	Low	8
Congress of NSSR and EBG Tamber et al. 2016 [19]	Conservative care	< 7 months	Anthropometry CVAI, 3D	Favourable in mod-severe cases or older children	Low	7
**Repositioning therapy**						
Shweikeh et al. 2013 [7]	Usual care	< 12 months	NR	Favourable	NR	2
Congress of NSSR and EBG Klimo et al. 2016 [18]	Helmet	NR	Cranial index, CVAI, photography, 3D analysis,	Unfavourable	Moderate	7
Congress of NSSR and EBG Klimo et al. 2016 [8]	Manual Therapy	NR	Cranial index, CVAI, photography, 3D analysis	Unfavourable	High	7
Congress of NSSR and EBG Klimo et al. 2016 [8]	Usual care	NR	Cranial index, CVAI, photography, 3D analysis	Favourable	Moderate to high	7
**Manual therapy**						
Bialocerkowski et al. 2005 [20]	Helmet		CVAI, parental perceptions	Inconclusive	Low	7
Parnell Prevost et al. 2019 [13]	Standard care	2 weeks	NR	Inconclusive favourable	Moderate	7
Congress of NSSR and EBG Baird et al. 2016 [8]	Positional advice	NR	Plagiocephalometry	Favourable	High	7
Congress of NSSR and EBG Baird et al. 2016 [8]	Positioning pillow	NR	Plagiocephalometry	Favourable	Moderate	7

**Table 5:** Summary of narrative analyses of treatments for Congenital Muscular Torticollis

Author	Follow-up time-point	Intervention Vs Control	C. Sp. PROM	Effect	Level of evidence	Amstar
**Stretching Exercises**						
Heidenreich et al. 2018 [25]	3 weeks where reported	Practitioner led stretching Vs Mixed (not reported)	Narrative	Favourable	Moderate	5
**Manual therapy**						
Brand et al. 2005 [24]	8 weeks	No studies identified for CMT/Plagio/KISS	No data available	N/A	N/A	3
Driehuis et al., 2019^a^ [4]	8 weeks	Spinal mobilization + physical therapy Vs physical therapy only	In both groups torticollis positively changed (IV: 80% improvement, C: 81.3%).	No significant difference between groups (p:0.85).	Very low quality	8
Parnell Prevost et al. 2019^a^ [13]	8 weeks	Manual Therapy + physio Vs physio only	Narrative	Inconclusive (unfavourable)	Moderate	7

^a^Parnell Prevost et al. 2019 and Driehuis et al. 2019 included the same study [26] but rated the level of evidence differently

One researcher independently extracted characteristics and data from the selected articles and a second reviewer checked the extraction. A third reviewer’s opinion was sought in cases of disagreement in the extraction process.

### Level of evidence

We used reported levels of evidence as published in the reviews and guidance and analysed these to indicate overall level of publication consensus on effectiveness and safety. The strength of the overall evidence was determined by the reviews and guidance evaluated as favourable, unfavourable or inconclusive with high, moderate or low certainty based on the quality of studies included.

## Results

**Fig. 1:**
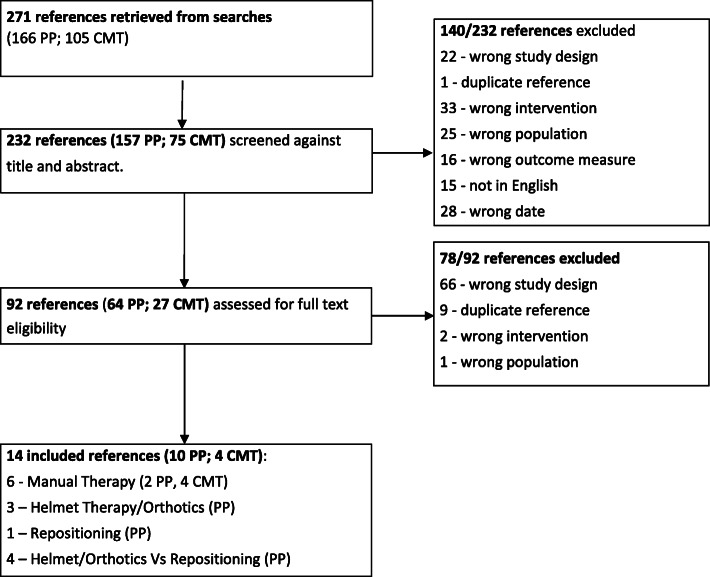
Flowchart of search process for the review

There were 232 studies selected for screening against title and abstract; of these 157 were for PP and 75 for CMT. Of the studies selected 140 were excluded leaving 92 for full paper review. A further 78 were excluded and 14 references were finally included in this systematic review. The final selection included 10 papers for PP and 4 for CMT. See Fig. 1.

There were 10 reviews in the final selection for positional plagiocephaly; 2 investigating effectiveness of manual therapy [8, 13], 3 for helmet therapy/orthotics [19, 21, 22], 1 for repositioning advice/therapy [18] and 4 papers which compared helmet therapy with repositioning [7, 11, 20, 23]. All of these reviews conducted a narrative analysis of their results.

There were 4 CMT reviews in the final selection which examined the effectiveness of manual therapy [10, 20, 23, 24]. However one review [24] searched for RCTs for Kinetic Imbalance for Sub-occipital Strain (KISS) syndrome which suggested that it is a component in both CMT and PP; they found no data to present on the treatment of this syndrome. Two of the reviews [10, 23] had data on one study only.

The characteristics of the final selection studies are shown in Tables 2 and 3.

### Outcomes

There were no meta-analyses in any of the studies reviewed for PP, but two for manual therapy with CMT [4, 24].

### Positional plagiocephaly

Six reviews reported on the effectiveness of helmet therapy for PP using a variety of methods for measuring cranial asymmetry. The control groups were either repositioning therapy or conservative care, where reported. The results in five out of the six reviews reported favourable outcomes for helmets but the quality of evidence was found to be low across all studies. Results of the reviews which reported on repositioning therapy were favourable in two studies when compared with usual care [7, 18] and unfavourable when compared with manual therapy or helmet therapy [18]. These studies were graded as moderate to high quality where reported. Manual therapy produced favourable outcomes when compared with repositioning therapy or standard care [8, 13] but inconclusive findings when compared with helmet therapy [20]. The quality of the studies within these reviews varied. See Table 4.

### Congenital muscular torticollis

Three reviews extracted data for the effectiveness of CMT treatments. One reported favourable results after 3 weeks of manual therapy (practitioner-led stretching) and the level of evidence was graded as moderate [25]. The two other reviews which reported on manual therapy for CMT each reviewed the same pilot study for data extraction: both intervention and control groups received paediatric physiotherapy, and the intervention group received additional spinal mobilization treatment (SMT). Although both intervention and comparator were forms of manual therapy, there was no significant difference in the outcome of the group with SMT in addition to physiotherapy. The systematic reviews weighted the level of evidence differently, very low and moderate [4, 13]. See Table 5.

### Adverse events

No adverse serious events were reported in any of the RCTs reviewed. However, 2 studies [4, 24] looked at adverse events in other types of studies and case report studies. Both found examples of serious adverse events. One case reported by Driehuis et al. (2019) described temporary quadriplegia following treatment in 4-month-old boy. The other case was reported in 2 studies [20, 23] which described death in a 3-month-old girl. While both of these adverse events were associated with cervical spine manipulation for CMT in one study [23], the other study [20] associated the death with the administration of Vojta, a physiotherapy technique, for KISS. See Table 6.

**Table 6:** Adverse event reporting

Authors	Adverse Events reported
**Positional Plagiocephaly**	
Bialocerkowski et al. 2005 [20]	Not reported
Goh et al. 2013 [21]	Not reported
McGarry et al. 2008 [22]	No serious harm was associated with cranial orthoses. Potential health risks such as skin irritation and breakdown due to excessive pressure, and heat and perspiration were noted. Occasional rashes on the infant’s skin caused by heat or reaction to materials may occur.
Paquereau et al. 2013 [23]	Re-positioning pillows on a mattress reduce the free mobility of the cephalic extremity of the infants. We must therefore wonder about the consequences of the restriction of these spontaneous movements which could have a facilitating role in the onset of the sudden infant death. The risk–benefit balance of the posterior positional plagiocephaly treatment should be taken into account.
Shweikeh et al. 2013 [7]	In the studies that reported adverse events, none were recorded.
Xia et al. 2008 [11]	Not reported
Parnell Prevost et al. 2019 [13]	None occurred in the RCTs. Not reported for observational studies
Congress of Neurological Surgeons Guideline: Positional Plagiocephaly 2016 [26]	Repositioning NR Physical therapy NR Helmet therapy NR
**CMT**	
Heidenreich et al. 2018 [25]	Not reported
Parnell Prevost et al. 2019 [13]	The RCT included does not mention of adverse events.
**Mixed PP and CMT**	
Brand et al. 2005 [24]	1 x case report: 1 x death following administration of Vojta therapy by physiotherapist for KISS
Driehuis et al. 2019 [4]	2 x case reports: 1 x temporary quadriplegia following cervical spine manipulation in 4-month-old boy and 1 x death following cervical spine manipulation in 3-month-old girl.

### Guidance

We found one guideline for positional plagiocephaly management that met our criteria and had a published guideline development procedure by the Congress of Neurological Surgeons Taskforce [26]. We were able to ascertain that it met at least 16 of the total AGREE II quality appraisal criteria (See Additional file 3). This guideline [27] was based on the systematic reviews by Klimo et al [22], Baird et al [5] and Tamber et al [21], all published in 2016 as part of the taskforce working group. The guideline recommended both physical therapy and repositioning as first line treatment followed by helmet therapy as a second line of treatment for infants with moderate to severe and persisting asymmetry. They recommended physical therapy above positioning pillows due to the risk of Sudden Infant Death Syndrome [26]. See Table 7.

**Table 7:** Summary Clinical Guideline recommendations for Positional Plagiocephaly

Recommendations	World Congress of Neurological Surgeons 2016^a^
Physiotherapy / Physical therapy	✓
Home exercise (e.g. passive stretching)	
Positioning (e.g. tummy time)	✓
Helmet/Orthotic therapy	✓
Repositioning pillow	✓
Education	✓

^**a**^**World:** Congress of Neurological Surgeons https://www.cns.org/sites/default/files/guideline-pdf/summary_with_recommendations_final_12.1.16.pdf Joint Guidelines Committee of the American Association of Neurological Surgeons (AANS) and the Congress of Neurological Surgeons (CNS) and American Academy of Pediatrics (AAP)

The national guidance for CMT by the American Physical Therapy Association met 21 of the 23 AGREE II quality criteria (See Additional file 4). The first line treatment recommendations were similar for both PP and CMT, i.e. parent education and support, positioning and tummy time, physical therapy including passive articulation and stretching. See Table 8.

**Table 8:** Summary Clinical Guideline recommendations congenital muscular torticollis

Recommendations	USA American Physical Therapy Association 2018^a^
Physiotherapy / Physical therapy	✓
Home exercise (eg passive stretching)	✓
Positioning (e.g. tummy time)	✓
Handling / feeding	✓
Education	✓
Botulinum	N/A (consider specialist referral if no response to PT treatment)
Surgery	N/A (consider specialist referral if no response to PT treatment)

^a^USA: American Physical Therapy Association (Kaplan et al. 2018) https://www.ncbi.nlm.nih.gov/pubmed/30277962

## Discussion

The results for treatments for PP indicated that repositioning therapy had moderate to high quality level of evidence showing a mix of favourable and inconclusive findings. Manual therapy interventions had moderate to high quality evidence showing favourable outcomes when compared with repositioning therapy and positioning pillows [8] but equivocal low-level evidence findings when compared with standard care and helmet therapy. Helmet therapy was favourable for head shape change but the quality of these studies was low, indicating that there was uncertainty around these findings. The effectiveness of helmet therapy compared to manual therapy was inconclusive [20].

The results for CMT showed that manual therapy in the form of practitioner-led stretching had moderate favourable evidence for increasing range of movement and there was low quality inconclusive evidence to support SMT in addition to physiotherapy. Caution is required when interpreting the evidence for manual therapy for CMT as it only relies on one underpowered pilot study with methods that were inappropriate to assess effectiveness [28].

It is a mixed and confusing batch of evidence which is compounded with poor standardisation of measurement criteria for both PP and CMT and a lack of available guidance. The first line treatment recommended in the guidance reviewed for both PP and CMT were: parent education and support, positioning / tummy-time and physical therapy. Parent education and support centred on reassurance that in the majority of cases positional preference and head shape can resolve over time and may be helped with repositioning and physical therapy (all guidance). If the education and guidance centres on the premise of natural resolution, the foundation for arguments for any kind of treatment or intervention seems limited. However some concern has been raised about severe and moderate cranial asymmetry (positional plagiocephaly and brachycephaly) and later cognitive and academic outcomes: direct causality was not suggested, the authors postulated that moderate / severe PP could be a marker for developmental risk, meaning that those who develop plagiocephaly may be at risk of developmental problems [14]. This implies a greater need to standardise measurement and definitions of mild, moderate and severe PP to determine which babies may benefit from extra care, monitoring and treatment.

Repositioning (including tummy time) and stretching regimes make intuitive sense when considering a mechanistic explanatory model for a treatment intervention. They may also have a high placebo element of effect because the parents are able to play an active role in helping to resolve the baby’s condition [24], This does not, however, negate the need of practitioner input as parents often require advice, guidance and support to understand how and when to do the stretching and repositioning. Potential problems identified with the home exercises, repositioning and tummy time included adherence and ‘critical dose’ needed to make a change. The clinician or therapist delivering advice, guidance and support to parents does not have to be limited to one profession, especially if this allows for more access to support, advice and care.

There were no serious adverse events documented in any RCTs included in the systematic reviews we reviewed for manual therapies, repositioning and helmets. One study mentioned serious adverse events reported in case studies but causality between the manipulation treatment and the adverse events were inconclusive [4]. Minor adverse events were noted with helmet therapy, such as some skin irritation and rashes which may affect comfort and the infants’ tolerance to wearing the helmet. Given this information it seems sensible to consider conservative treatments as a first line option unless otherwise indicated.

There was also a suggested issue with positioning pillows and the potential associated risk of infants not sleeping on their back and sudden infant death syndrome. Several studies concluded that despite the moderate and favourable beneficial effects on head shape and symmetry with the use of positioning pillows, it did not outweigh the risk of sudden infant death syndrome [8, 24]. The Congress of Neurological Surgeons’ guidance recommended that manual therapy and repositioning be used rather than positioning pillows [8].

A limitation of reviews of reviews is that many of the same studies are repeatedly analysed, however we were able to compare the interpretation of findings between the different authors using the same studies. We noted that the authors consistently identified issues with non-standardised diagnostic criteria to classify levels of severity of PP and CMT and how the outcomes were measured, for example cranial asymmetry and/or cervical range of movement. We did not analyse these factors in this review, nor did we consider treatments for more severe cases of PP and CMT when an infant has clinical development or thriving issues (rather than observable mild cosmetic issues) that may indicate surgical interventions or botulinum. In addition, we did not review studies to fully comprehend meaningful change to the infants and their parents.

A strength of this review was that we compared synthesised evidence with the guidance and we noted that guideline recommendations were not fully grounded in high quality favourable evidence. We would concur with others [24] that there is some favourable, albeit low-quality evidence, of some benefit for manual therapy in the form of practitioner-led stretching for CMT only and repositioning for both PP and CMT with no reported serious adverse events, warranting that these should be considered in the first instance.

Further research is needed to understand when, and if, to intervene and the optimal stages of interventions for what kind of benefit, risk or discomfort to the infants. At present there is not enough robust data or evidence to fully inform guideline development. The most comprehensive attempt by the Congress of Neurological Surgeons [26] illustrated what a difficult and varied task it was to develop the guidance. They did three different systematic reviews to try and fully comprehend the literature surrounding PP.

## Conclusions

Manual therapy for PP showed favourable outcomes when compared with repositioning therapy but equivocal low-level evidence when compared with helmet therapy. The results for CMT showed that manual therapy in the form of practitioner-led stretching had moderate favourable evidence for increasing range of movement but this evidence only relies on one underpowered pilot study. More well powered effectiveness and efficacy studies are needed.

Distinguishing between superiority of treatments was difficult due to the lack of standardised measurement systems, the variety of outcomes and limited high quality studies. There is still a need to have an accepted classification system for diagnosing and describing grades of PP and CMT to fully investigate when and if therapy is warranted and what change is a meaningful change. The type of treatment appropriate for the infants and the duration and dose of treatment is yet to be clearly determined.

Overall physical therapy such as stretches and/or exercises, repositioning and tummy-time may benefit some infants. Education, guidance and support is likely to reassure and help parents. Advice and guidance can be given by a variety of health care professional’s but clearly clinical training is necessary to ascertain whether the infants are healthy and thriving with no underlying pathologies before conservative approaches and/or treatments are recommended.

## Supplementary information

**Additional file 1: Appendix 1.** Search string examples

**Additional file 2: Appendix 2.** AMSTAR Quality Appraisal of Included Studies

**Additional file 3: Appendix 3.** Quality appraisal of guidance AGREE II Score for PP

**Additional file 4: Appendix 4.** Quality appraisal of guidance AGREE II Score for CMT
