# Supplementary material for: The effectiveness and safety of conservative interventions for positional plagiocephaly and congenital muscular torticollis: a synthesis of systematic reviews and guidance
Source: Chiropr Man Therap. 2020 Jun 11;28:31. doi: 10.1186/s12998-020-00321-w (PMC7288527; doi:10.1186/s12998-020-00321-w)
Supplement: Supplementary file 1 — Additional file 1: Appendix 1. Search string examples [file 12998_2020_321_MOESM1_ESM.docx]

**Appendix 1. Search string examples**

PubMed

("therapeutics"[MeSH Terms] OR "therapeutics"[All Fields] OR "treatments"[All Fields]) AND ("Congenital torticollis"[Supplementary Concept] OR "Congenital torticollis"[All Fields] OR "congenital torticollis"[All Fields])

("therapeutics"[MeSH Terms] OR "therapeutics"[All Fields] OR "treatments"[All Fields]) AND ("plagiocephaly"[MeSH Terms] OR "plagiocephaly"[All Fields])

“nonsyndromic craniosynostosis”, “nonsynostotic cranial deformity”, “positional deformity”. “nonsynostotic posterior plagiocephaly”, and “positional plagiocephaly”

Embase

('congenital muscular torticollis'/exp OR 'congenital muscular torticollis') AND ([cochrane review]/lim OR [systematic review]/lim OR [meta analysis]/lim) AND [1999-2019]/py

('plagiocephaly'/exp OR plagiocephaly) AND ([cochrane review]/lim OR [systematic review]/lim OR [meta analysis]/lim) AND [1999-2019]/py

MANTIS

‘Congenital muscular torticollis’ OR torticollis AND treatment AND review

‘Cranial asymmetry’ OR plagiocephaly AND treatment AND review
