# Supplementary material for: The effectiveness and safety of conservative interventions for positional plagiocephaly and congenital muscular torticollis: a synthesis of systematic reviews and guidance
Source: Chiropr Man Therap. 2020 Jun 11;28:31. doi: 10.1186/s12998-020-00321-w (PMC7288527; doi:10.1186/s12998-020-00321-w)
Supplement: Supplementary file 2 — Additional file 2: Appendix 2. AMSTAR Quality Appraisal of Included Studies [file 12998_2020_321_MOESM2_ESM.docx]

| **Appendix 2 – AMSTAR Quality Appraisal of Included Studies**  (Shaded cells represent those graded in this study – range 0 – 8; Yes = 1, No = 0, Partial Yes = 0.5) | | | | | | | | | | | | | | | | | |
| --- | --- | --- | --- | --- | --- | --- | --- | --- | --- | --- | --- | --- | --- | --- | --- | --- | --- |
| **Positional Plagiocephaly** | | | | | | | | | | | | | | | | | |
| Author | 1 | 2 | 3 | 4 | 5 | 6 | 7 | 8 | 9a | 9b | 10 | 11 | 12 | 13 | 14 | 15 | Amstar score |
| Bialocerkowski et al, 2005 | Yes | Partial Yes | Yes | Yes | Yes | Yes | No | Yes | ION | Yes | No | NMC | NMC | Yes | NMC | No | 7 |
| Goh et al, 2013 | Yes | Partial Yes | Yes | No | No | No | No | No | ION | No | No | NMC | NMC | No | NMC | Yes | 2 |
| McGarry et al, 2008 | Yes | Partial Yes | Yes | Partial Yes | Yes | No | No | Yes | ION | Yes | Yes | NMC | NMC | No | NMC | No | 4.5 |
| Paquereau, J. 2013 | Yes | Partial Yes | Yes | Partial Yes | No | No | No | Yes | ION | No | No | NMC | NMC | No | NMC | Yes | 3.5 |
| Shweikeh et al, 2013 | Yes | Partial Yes | No | No | No | No | No | Yes | No | No | No | NMC | NMC | No | NMC | No | 2 |
| Xia et al, 2008 | Yes | Partial Yes | Yes | Yes | Yes | Yes | No | Yes | ION | Yes | No | Yes | Yes | Yes | Yes | Yes | 8 |
| Parnell Prevost et al, 2019 | Yes | Partial Yes | Yes | Yes | Yes | Yes | No | Yes | Yes | Yes | No | NMC | NMC | No | NMC | Yes | 7 |
| Baird 2016 | Yes | Yes | Yes | Yes | Yes | Yes | No | Yes | Yes | IOR | No | No | No | No | No | Yes | 7 |
| Kilmo 2016 |  |  |  |  |  |  |  |  |  |  |  |  |  |  |  |  |  |
| Tamber 2016 |  |  |  |  |  |  |  |  |  |  |  |  |  |  |  |  |  |
| **Congenital Muscular Torticollis** | | | | | | | | | | | | | | | | | |
| Brand et al, 2005 | Yes | Partial Yes | Yes | Yes | No | No | No | No | No | IOR | No | Yes | Yes | Yes | No | No | 3 |
| Heidenreich et al, 2018 | Yes | Partial Yes | Yes | Yes | No | Yes | No | Yes | Yes | IOR | No | NMC | NMC | No | No | No | 5 |
| Parnell Prevost et al, 2019 | Yes | Partial Yes | Yes | Yes | Yes | Yes | No | Yes | Yes | Yes | No | NMC | NMC | No | NMC | Yes | 7 |
| Driehuis et al, 2019 | Yes | Yes | Yes | Yes | Yes | Yes | Yes | Yes | Yes | Yes | No | Yes | Yes | Yes | Partial Yes | Yes | 8 |

**IOR –** Included only RCT’s**; ION –** Included only NRSI’s**; NMC –** No meta-analysis conducted
