# Supplementary material for: The effectiveness and safety of conservative interventions for positional plagiocephaly and congenital muscular torticollis: a synthesis of systematic reviews and guidance
Source: Chiropr Man Therap. 2020 Jun 11;28:31. doi: 10.1186/s12998-020-00321-w (PMC7288527; doi:10.1186/s12998-020-00321-w)
Supplement: Supplementary file 4 — Additional file 4: Appendix 4. Quality appraisal of guidance AGREE II Score for CMT [file 12998_2020_321_MOESM4_ESM.docx]

**Appendix 4 Quality appraisal of guidance AGREE II Score for CMT**

| **Domain** | **Item** |  |
| --- | --- | --- |
|  |  | **American Physical Therapy Association 2013** |
| Scope and purpose | 1. The overall objective(s) of the guideline is (are) specifically described. | Y |
|  | 1. The health question(s) covered by the guideline is (are) specifically described. | Y |
|  | 1. The population (patients, public, etc.) to whom the guideline is meant to apply is specifically described. | Y |
| Stakeholder involvement | 1. The guideline development group includes individuals from all the relevant professional groups. | Y |
|  | 1. The views and preferences of the target population (patients, public, etc.) have been sought. | Y |
|  | 1. The target users of the guideline are clearly defined. | Y |
| Rigor of development | 1. Systematic methods were used to search for evidence. | Y |
|  | 1. The criteria for selecting the evidence are clearly described. | Y |
|  | 1. The strengths and limitations of the body of evidence are clearly described. | Y |
|  | 1. The methods for formulating the recommendations are clearly described. | Y |
|  | 1. The health benefits, side effects and risks have been considered in formulating the recommendations. | Y |
|  | 1. There is an explicit link between the recommendations and the supporting evidence. | Y |
|  | 1. The guideline has been externally reviewed by experts prior to its publication. | Y |
|  | 1. A procedure for updating the guideline is provided. | Y |
| Clarity of presentation | 1. The recommendations are specific and unambiguous. | Y |
|  | 1. The different options for management of the condition or health issue are clearly presented. | Y |
|  | 1. Key recommendations are easily identifiable. | Y |
| Applicability | 1. The guideline describes facilitators and barriers to its application. | Y |
|  | 1. The guideline provides advice and/or tools on how the recommendations can be put into practice. | Y |
|  | 1. The potential resource implications of applying the recommendations have been considered. | Y |
|  | 1. The guideline presents monitoring and/ or auditing criteria. | Y |
| Editorial independence | 1. The views of the funding body have not influenced the content of the guideline. |  |
|  | 1. Competing interests of guideline development group members have been recorded and addressed. |  |
| Overall Guideline Assessment | The overall quality of this guideline. | **High**  **21/23** |
